# RF Coil Setup for ^31^P MRSI in Tongue Cancer *in vivo* at 7 T

**DOI:** 10.3389/fneur.2021.695202

**Published:** 2021-11-02

**Authors:** Ria Forner, Kyungmin Nam, Klijs J. de Koning, Tijl van der Velden, Wybe van der Kemp, Alexander Raaijmakers, Dennis W. J. Klomp

**Affiliations:** ^1^Division of Imaging and Oncology, University Medical Centre (UMC) Utrecht, Utrecht, Netherlands; ^2^Ceresensa Inc., London, ON, Canada; ^3^Surgery, University Medical Centre (UMC) Utrecht, Utrecht, Netherlands; ^4^Division of Biomedical Image Analyses, Technical University Eindhoven (TU/e), Eindhoven, Netherlands

**Keywords:** ^31^P MRSI, tumour, ^31^P, RF coil, tongue, 7 T, UHF

## Abstract

Surgery for tongue cancer often results in a major loss in quality of life. While MRI may be used to minimise the volume of excised tissue, often the full tumour extent is missed. This tumour extent may be detected with metabolic imaging. One of the main reasons for the lack of metabolic information on tongue cancer would be the absence of an x-nuclear coil with the tongue as a focus target. Metabolic MRI through ^31^P MRSI is known as a powerful tool to non-invasively study elevated cell proliferation and disturbed energy metabolism in tumours. Severe magnetic field non-uniformities are inherently caused by the substantial difference in magnetic susceptibilities of tissue and air in the mouth and its environs. Despite this, the wide chemical shift dispersion of ^31^P could still facilitate precise detection of the cell proliferation biomarkers, phospomonoesters and diesters, as well as energy metabolites ATP, inorganic phosphate, and phosphocreatine potentially mapped over the tongue or tumour *in vivo*. In this study, we present the first ^31^P MRSI data of the human tongue *in vivo* from healthy volunteers and a patient with a tongue tumour at 7 T MRI using a ^1^H 8-channel transceiver setup placed inside a body ^31^P transmitter, which is able to get a uniform excitation from the tongue while providing comfortable access to the patient. In addition, a user-friendly external ^31^P receiver array is used to provide high sensitivity (80%) comparable to an uncomfortable inner mouth loop coil positioned on the tongue. The primary aim is the demonstration of ^31^P metabolite profiles in the tongue and the differences between healthy and malignant tissue. Indeed, clear elevated cell proliferation expressed as enhanced phosphomonoesters is observed in the tumour vs. the healthy part of the tongue. This can be performed within a total scan duration of 30 min, comparable to clinical scans, with a spatial resolution of 1.5 cm for the 10-min ^31^P MRSI scan.

## Introduction

Amongst all intra-oral cancers, tongue cancer has the highest incidence, occurring in about 30% of all intra-oral cancer cases (1). The incidence rates vary globally, with high incidence rates in India and parts of Europe (1). While with surgery, the 5-year survival rates are on average 50–60% (2), the surgery itself has a significant impact on quality of life. A retrospective analysis in our centre showed that, in line with other literature (3), 84% of the resected specimens had inadequate resection margins (i.e., tumour cells <5 mm from the boundaries) (4). Inadequate resection margins are associated with low survival and are therefore an indication to apply postoperative treatment in the oral cavity, i.e., re-resection and radiotherapy (5). Postoperative treatment has been reported in 35% of the oral cancer patients in our centre (15.5% re-resections and 19.5% radiotherapy), part of which could have been prevented by better margin control (6). Particularly, postoperative intraoral radiation may affect the quality of life of our patients due to significant morbidity and (oral) discomfort, including xerostomia, mucositis, fibrosis, and osteoradionecrosis.

To improve survival in patients with early tongue cancer, understanding the primary tumour extent is indispensable. In clinical practise, the extent of the primary oral cancer is evaluated by physical examination and imaging, e.g., MRI. Although MRI is regarded as the preferred imaging modality in OSCC (oral squamous cell carcinoma), it frequently underestimates as well as overestimates the extent of the tumour (7, 8). Inflammation surrounding the tumour could mimic or blur the boundaries that are observed in images (8).Therefore, improvements in MRI techniques are warranted to improve delineation of OSCC and subsequently decrease the rate of inadequate surgical margins (9). Conventional MRI makes use of extra-oral receive coils. Alternative MR imaging techniques, like MRI with an intra-oral coil, have been pioneered to assess tumour extent better. With an intra-oral coil, the highest sensitivity could be obtained for high-resolution anatomical MRI of the tongue (10). However, even in *ex vivo* MRI measurements where artefacts due to motion and field non-uniformity are minimal, surgical margins could not be assessed accurately in the anatomical MR images when compared to whole mount histopathology (11).

An alternative to imaging tumour extent based on anatomical characterisation is to investigate other image contrast mechanisms. Rather than observing water or its MR relaxation properties, one can also observe the energy and cell proliferation metabolism with MR, known to be substantially altered in tumour tissue. When observing ^31^P MR spectroscopic imaging, one can reveal energy metabolites like PCr (phosphocreatine), ATP (adenosine tri-phosphate), Pi (inorganic phosphate), or cell proliferation markers like PMEs (phosphomonoesters) [PC (phosphocholine) and PE (phosphorylethanolamine)] and PDEs (phosphodiesters) [GPE (glycerophosphorylethanolamine) and GPC (glycerophosphorylcholine)] (12, 13). Should the disturbed metabolic profile be visible post-surgery, it would indicate non-removal of tumour.

Relative proportions of metabolites indicate better response to different treatment plans. For instance, one may consider chemotherapy in cases where cell proliferation is high (i.e., high PME levels). However, the concentration of these metabolites is four orders of magnitude lower than water *in vivo*, while the gyromagnetic ratio of ^31^P is also 2.5 times lower than that of ^1^H. Consequently, for obtaining a comparable signal-to-noise ratio (SNR), the pixel size needs to be increased from sub-millimetre to more than a centimetre, i.e., increasing pixel volume by three to four orders of magnitude. Moving up in field strength from 3 to 7 T should further increase SNR more than two-fold (14).

It has been previously demonstrated that for ^31^P MRSI (magnetic resonance spectroscopy imaging) in breast cancer even in Schmitz et al. (15), a small tumour of 6 mm in size, altered PME (PE+PC)/PDE (GPE+GPC) levels could be observed when compared to healthy tissue despite partial volume effects and even in poorly B_0_ shimmed areas (16). Due to the presence of sinus cavities, the B_0_ homogeneity is less than ideal, which challenges the use of diffusion-weighted imaging (17). ^31^P MRSI was considered possible as with this, there are no artefacts from highly abundant water or lipid signals, and the chemical shift dispersion of ^31^P is so large that even in poor field uniformity, the peaks (PME and PDE) do not overlap. So, ^31^P MRSI may be a good technical solution to the substantial B_0_ shimming challenges exacerbated at 7 T, especially in the oral cavity due to the close proximity of air-filled cavities.

While dedicated receive-only tongue coils have been demonstrated for proton imaging *in vivo* at 3 T in combination with a body transmit coil, the required setup for ^31^P is considered more complex. This complexity may be the reason for the absence of ^31^P MRSI data of tongue tumours in literature. Local transmit-receive coils can be used for ^31^P MRSI, but their non-uniform transmit field requires the use of adiabatic RF pulses. The resultant high levels of RF power deposition may violate SAR guidelines or compromise scan duration. Recently, it was shown that rather than a ^1^H body coil, a ^31^P body coil could be integrated into a 7-T MR system, using dipole antennas for ^1^H transmit (18). This way, the ^31^P setup could be substantially simplified, avoiding the need for adiabatic RF pulses and thus maintaining SAR guidelines within relatively short scan times. While other ^31^P head coils (19, 20) have been reported in literature, these were optimised for brain imaging.

With the presence of such a built-in transmit coil for ^31^P and an array of dipole antennas for ^1^H imaging, the anatomy of the tongue would allow close proximal positioning of ^31^P receive coils. An intra-oral coil placed supra-lingually would be ideally situated for maximum signal reception from the tongue. Unfortunately, there are two drawbacks to such a coil: (1) patients with painful tumours may not tolerate such a device for long periods, and (2) making the surface of the coil perpendicular to the static magnetic field and thus maximising signal requires holding the neck in an uncomfortable position for the duration of the scan protocols. So, keeping the subject's comfort in mind, an external coil would be more suitable.

In our study, we demonstrate the feasibility of ^31^P MRSI of tongue cancer at 7 T. We present the design of a three-channel external ^31^P Rx (receive) array, combined with a distal 8 channel TxRx (transceive) dipole array for ^1^H excitation and reception placed inside a ^31^P full-body Tx birdcage coil. In addition, we show that the SNR of the ^31^P MRSI obtained in the tongue of healthy subjects when using the external array is comparable to the use of an intra-oral coil, highlighting the practically uncompromised added comfort of the external array. Finally, we demonstrate well-visible and distinguished signals from PME, PDE, PCr, Pi, and ATP with our setup mapped over the tongue in a patient with tongue cancer.

## Methods

### Hardware

**Figure 1:**
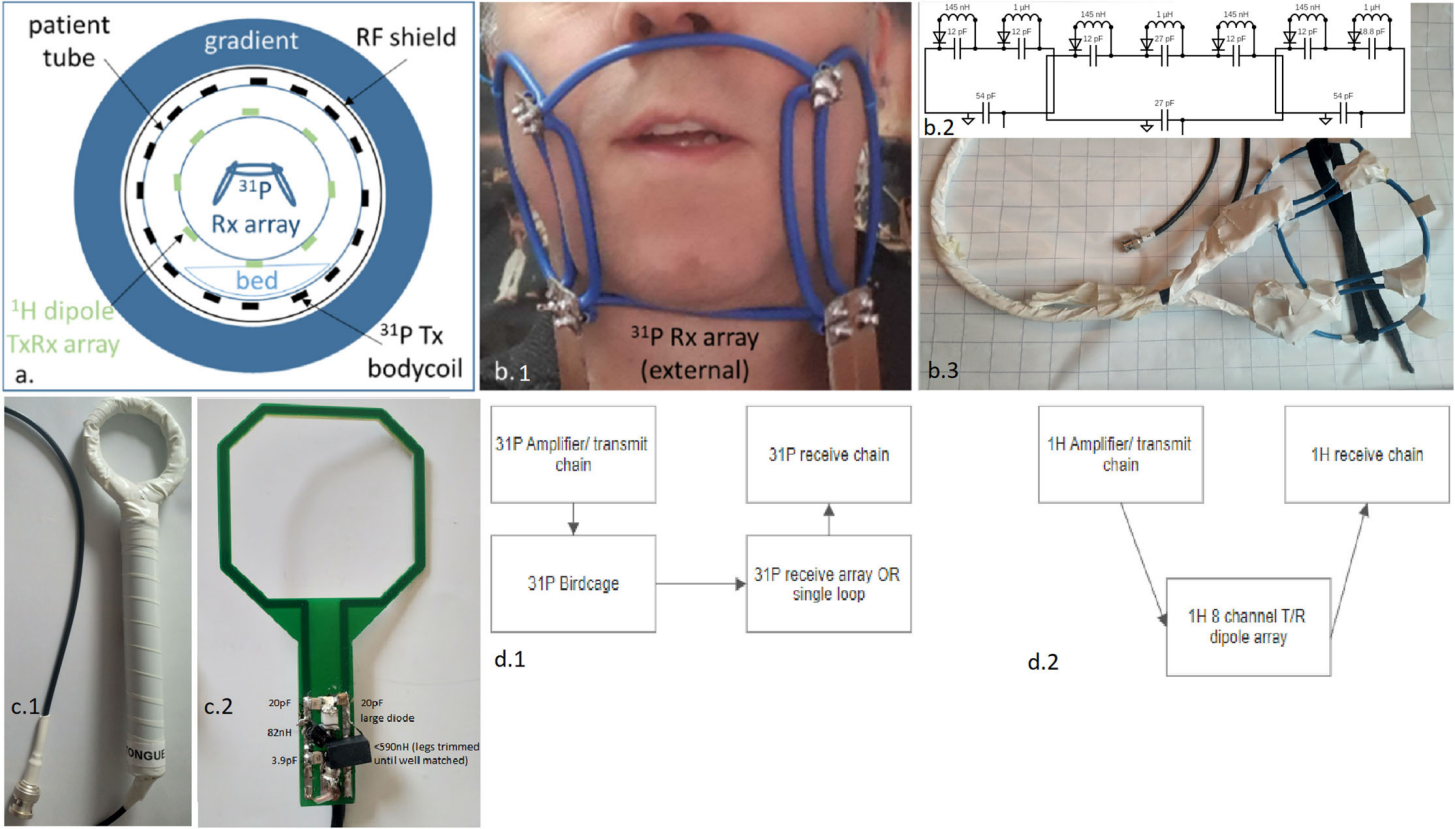
Representation of the used complete RF setup expressed in a schematic overview **(a)**, as a photo when positioned on a subject, zoomed onto the external three-channel receiver array **(b1)**, a circuit schematic of the three-channel array (representative only—not to scale) with all component values **(b2)**, photo of three-channel array with insulation **(b3)**, and the reference intra-oral/ insert coil; shown with insulation present **(c1)**, and without insulation present and with component values indicated on photo **(c2)**, and the block schematic of the ^31^P **(d1)** and the ^1^H transmit and receive chains **(d2)**.

The ^31^P and ^1^H RF coil setup is composed of an embedded ^31^P volume transmitter, an eight-channel ^1^H TxRx dipole array mounted on a wide access cylinder, and a three-channel ^31^P receiver array constructed as a face mask (see Figures 1a,b for visual representation). The ^31^P volume transmitter is a quadrature birdcage (21) driven with a two-channel (2 × ) 18-kW RF amplifiers (Analogic 8137, Boston Massachusetts). The ^31^P body birdcage (inside bore) is a pre-existing coil. All other coils mentioned, including the proton TxRx array, were purpose-built for this specific project.

#### ^1^H TxRx Dipole Array

The ^1^H dipole array is based on the fractionated dipole design (22) and mounted on a 30-cm diameter Plexiglas former. The fractionated antennas have a length of 32 cm and have self-reflection parameters of −9 dB (worst case) when loaded with the human head. The dipole array was connected to the multi-transmit port of the 7 T MR system (Philips Healthcare, Best) that includes eight transmit/receive switches (duplexers) and can drive up to 2 kW per channel of RF peak power.

#### ^**31**^P External Receive Array

The external three-channel ^31^P receiver array is composed of three partially overlapping loops, bent to approximate the curvatures of a typical face mask (see Figure 1b). The array is 24 cm across and has a width of 10 cm so as to adequately cover the field of view. The central, biggest loop has a diameter of about 12 cm, while the other two loops are about 9 cm each. Rigid 6-mm^2^ isolated wire was used as conductors for the coil element. Due to the rigidity of the loops, a mechanical frame was not needed. This allows for an open albeit electrically insulated (see Figure 1 photo with and without insulation) frame such that the setup minimally impedes the respiration of the subject and reduces discomfort.

The central loop is positioned under the nose and reaches under the chin. This loop is opened in four locations, each connected to a small printed circuit board (PCB) with a tuning capacitor and detuning circuitry, as a consequence of the design and the four points of overlap with the neighbours. A minimum of two breaks were used to accommodate the segmenting capacitors for taking into account the wavelength and the total conductor length.

The remaining two loops are each opened in the two locations that match the crossing of conductors to provide the partial overlap and share the central loop's PCBs. The two PCBs located to the chin's left and right are equipped with matching capacitors and a wire-wound coaxial cable tuned with a capacitor to 120 MHz to ensure common mode current rejection (i.e., act as cable traps, not shown in electrical diagram, but embedded in PCB 1 and 2 shown in Figure 1).

Each Rx coil is tuned to the ^31^P Larmor frequency of 120 MHz and matched to 50 Ω when loaded with the face of an adult male and female. The ratios of unloaded to loaded Q factors for these loops are 4, when loaded with a human head. The three cables from the array are connected to an interface box that contains the preamplifiers, a detuning malfunction check and interface to the MR system. The scanner checks the DC current to bias the PIN diode. Below a certain threshold, the scanner will abort or prevent scanning. Preamplifier decoupling was not implemented due to the inherent decoupling *via* overlap and strong tissue loading.

#### Comparison to Single Internal Loop

To compare the performance of the external array to a closely positioned internal coil, a single loop coil of 5 cm × 6 cm was designed from a PCB, where the capacitors and detuning circuit are located just outside the mouth and the PCB is positioned directly on top of the tongue (Figure 1c). A 5-mm spacer enclosed in a latex housing was used to isolate the opened PCB from the tongue. Given the large wavelength at 120 MHz, differences in load sensitivity were not seen along the loop length. The coil was tuned to 120 MHz and matched to 50 Ω, and connected to the same interface as used for the external array. A ratio of 10 was seen for the unloaded and loaded Q factors.

### RF Safety Assessment

#### ^31^P Body Birdcage

The design and dimensions of the ^31^P body coil at 7 T are based on a classical 3 T body coil for ^1^H in clinical MRI that operates on 128 MHz. Therefore, SAR settings from well-established 3 T MRI systems can be reapplied to the ^31^P body coil, assuming all transmitted power to be absorbed by the subject, as described by Löring et al. (18).

#### ^1^H TxRx Dipole Array

The SAR of the ^1^H dipole array was simulated in circularly polarised mode (Sim4Life, Zurich MedTech, Switzerland) using “Duke” as a model to determine the maximum average power based on peak local SAR and global SAR. As the ^1^H setup is mostly used for brain imaging, the conventional quadrature drive would not be optimal when applied to the tongue. Since we intend to use the ^1^H setup mainly for background imaging and B_0_ shimming, we took a conservative approach where the per-channel power constraints were based on levels when driving the array with arbitrary phase settings and uniform power distribution over the eight elements (23). Considering that the head presents a different loading of the transmitter than the body for ^31^P, and the performance of a coil (matching and tuning included) depends on loading, an *in vivo* flip-angle recalibration was performed with a human subject positioned with the tongue in the iso-centre of the magnet.

#### ^31^P External Receive Array

The ^31^P local receiver coils are equipped with detuning circuits that prevent focusing of the RF power deposition in close proximity of the receiver coils. Bench tests (S_12_) were performed to verify the performance of detuning by moving a small pick-up probe over the conductor of the detuned receiver coil in the presence of an intrinsically uniform transmit field (using <1 dB as acceptance criteria). The procedure to test residual coupling is as follows: we start by detuning the Rx coil. Now, when the pickup probe (measuring the Tx field) is moved across the coil conductor (at the same distance as the closest load in the use case), half the difference between the lowest and highest S_21_ values picked up by this coil quantifies the coupling. Less than 1 dB corresponds to <10% B_1_ disturbance, which is in alignment with the scanner manufacturer specifications for receiver coils. The pickup loop is 1 cm in diameter.

As a secondary safety measure, a potential malfunction of the detuning circuitry was tested, in line with traditional ^1^H commercially available receiver coils: The MRI system checks for the ability to drive a direct current (DC) within set boundaries through the detuning circuit and prevents scanning if the current exceeds specifications. The malfunction detection circuit was tested by deliberately opening the DC circuit during a scan and verifying that the scan immediately aborts.

In terms of the loading, proximity and hence the SAR *in vivo*, the internal loop coil has the highest risk of violating SAR requirements. As a result, the external coil was not as rigorously tested due to the inherently insignificant risks.

#### ^**31**^P Internal Receive Loop

Finally, for the insert coil, a B${}_{1}^{+}$ map at the ^1^H frequency was obtained, once with and once without the presence of the receiver coil to verify the absence of local B${}_{1}^{+}$ alterations caused by the receiver (24).

### Subjects

A total of three volunteers were enrolled in this study. All gave written informed consent and the protocol development was approved by the local ethical committee. Moreover, the non-commercial ^31^P and ^1^H transmit setup was described in an investigation medical device dossier, which was approved by an independent auditing board. Subject 1 (female, 30 years) was scanned once with the internal coil (Figure 1c) and once with the external array (Figure 1b). Subject 2 (male, 46 years) was scanned with the external setup. Finally, subject 3, a patient with tongue cancer (male, 68 years), was scanned with the external setup. Subjects were instructed to avoid movement for the ^1^H scans as well as the 3D CSI. They were recommended to touch the tip of their tongue to the front teeth, although considering the presence of painful tumours, this may not have been practicable for all.

### Scan Protocol

**Figure 2:**
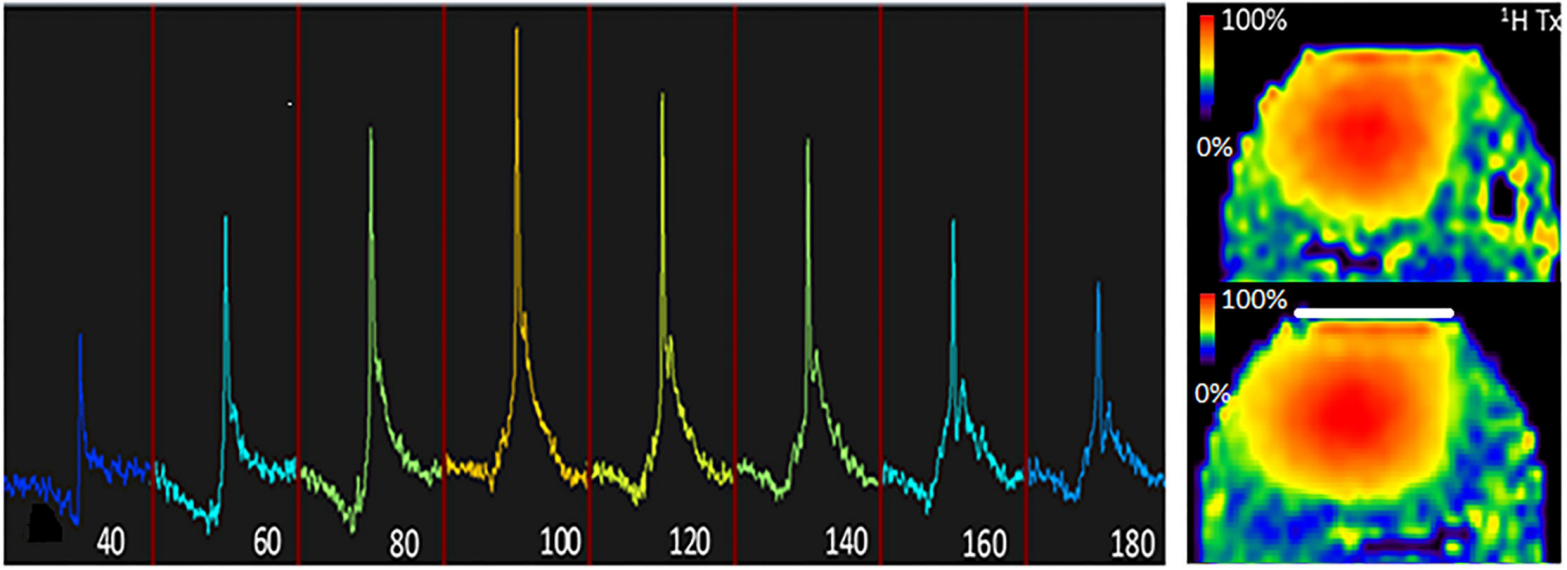
B${}_{1}^{+}$ characterisation of the setup. Left, ^31^P flip angle series of the tongue using a nominal flip angle of 40° through 180° at TR = 30 s when using the ^31^P volume coil as transmitter and the ^31^P local receiver as detector, indicating that the actual flip angle (or B${}_{1}^{+}$) is 10% less than nominal. Right, ^1^H B${}_{1}^{+}$ maps were obtained with the dipole array, once without the presence of the insert coil (top) and once with the presence of the insert coil (bottom). Note that the presence of the insert coil does not significantly (<10% difference) affect B${}_{1}^{+}$ for ^1^H.

The assessment of potential coupling of the ^1^H antennas to the ^31^P insert coil was verified by phantom measurement in a 10-cm Perspex sphere filled with physiologic salt (0.9% NaCl). This sphere contains two additional smaller solution-filled spheres, the first containing 200 mM of Pi and the other containing 50 mM each of PC, PE, and GPC. The reader's attention is drawn to the fact that this was done merely to check the starting values for the *in vivo* setup and that a flip angle series was further performed *in vivo* for quantification. A 3D B${}_{1}^{+}$ map (25) was obtained for ^1^H with a spatial resolution of 2 × 2 × 10 mm, with and without the presence of the insert coil for comparing the difference in the B${}_{1}^{+}$ map as shown in Figure 2.

In one subject, the ^31^P flip angle series was obtained using a rectangular RF pulse with a repetition time of 10 s and increasing B_1_ amplitude up to the maximum available power of the RF amplifier. The carrier frequency was set to the PCr resonance, and the nominal flip angle ranged from 40° until 180° with a 20° interval, using the volume transmitter. The local ^31^P insert receiver was used to localise the signal to the tongue.

A multi-slice image in the rapid gradient echo sequence (FFE) was acquired to determine the tongue's location within the head of the subject. The field of view was set to 224 × 224 × 82.5 mm and a voxel size of 2.33 × 2.33 × 5.5 mm. The flip angle was set to 10 s, with a TE of 1.25 ms, TR of 30 ms, and a single average with a slice thickness of 5 mm. For the first-order B_0_ shimming, a 3D B_0_ map was obtained (ΔTE = 1 ms). The FWHM (full width of the peak at half the maximum amplitude) linewidth of the distribution of B_0_ offset in the tongue area is reported, after which the carrier frequency is fixed to water for ^1^H and by fixed ratio automatically to PCr for ^31^P.

The 3D ^31^P MRSI data were acquired in a field of view of 224 × 224 × 150 mm with a spatial resolution of 15 × 15 × 15 mm isotropic with a 5-kHz bandwidth. The acquisition window was set to 50 ms (i.e., spectral 20 Hz resolution) to allow a short TR of 57 ms, TE of 0.61 ms, and an optimal flip angle of 10°. The maximum B${}_{1}^{+}$ was 6 μT. Hamming weighted acquisition was applied with 50 averages of the centre parts of k-space resulting in a total scan time of 10 min for the ^31^P MRSI scan. Afterwards, the reconstruction was performed following noise de-correlation, averages, spatial Hamming filtering, channel combination [whitened singular value decomposition (WSVD) (26)], 20 Hz spectral line broadening, fixed first-order phase correction, and automated zero-order phase correction using the CSIgui toolbox (27). The total scan session, including the proton scans, adds up to a total scan duration of at least 30 min, sometimes stretching up to 60 min in case of repeated scans due to subject motion.

## Results

### ^31^P Receive Performance on the Bench

Bench-top S_12_ measurements confirmed that in the detuned state, the receivers caused <1 dB of B_1_ field disturbance in close proximity of the conductors. When comparing the S_12_ between a perfectly aligned small pickup probe positioned on top of the centre of the tongue to each of the loops of the external array, about 6 dB (centre coil) or 7 dB (left and right coil) of loss was observed when compared to the internal coil.

#### External Array

All subjects could comfortably be positioned in the setup when using the external array. The insert loop was considered very uncomfortable as it causes substantial accumulation of saliva, and swallowing is complicated and influences coil placement. Moreover, the head needs to be bent forward to ensure an orthogonal orientation of the loop with respect to the main field of the magnet to ensure highest SNR, which was experienced as uncomfortable. Nonetheless, one subject could successfully complete the ^31^P MRSI study with the insert coil as the receiver in place.

**Figure 3:**
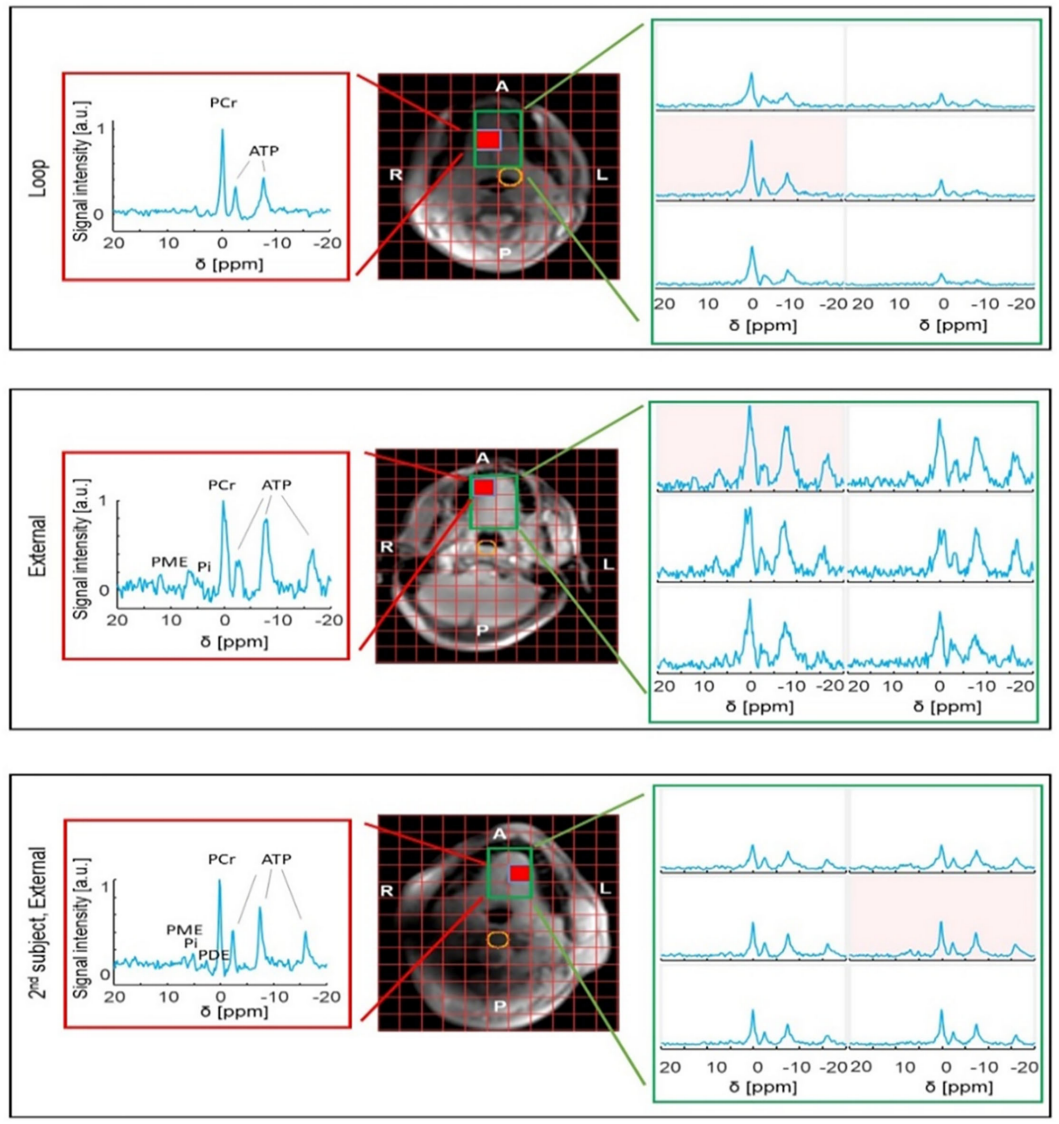
^31^P MRSI results obtained from the tongue of healthy volunteers using either the internal loop coil (central voxel position marked in yellow) (top) or external array (middle and bottom). Note that the SNR is substantially variable between subjects. The red voxel shows the best-case SNR for the slice. The variable proton signal is due to the short wavelength of ^1^H at 7 T in water (phantom).

When comparing the ^31^P results from the three healthy subjects, it can be observed that the SNR is substantially variable; however, in all voxels from the tongue, resonances of PCr, ATP, PME, and/or PDE and Pi could be observed (Figure 3).

**Figure 4:**
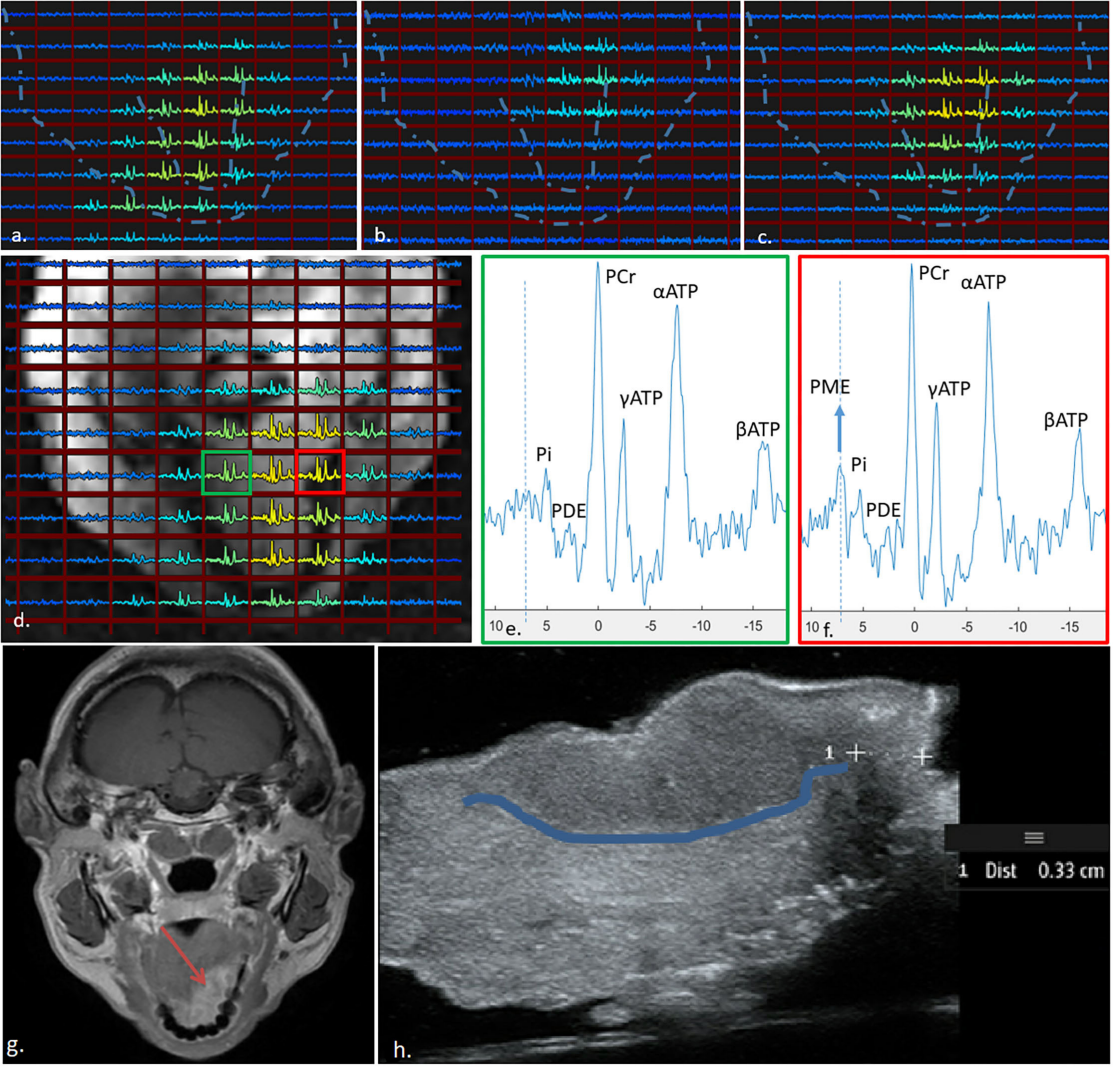
^31^P MRSI results obtained from the tongue of a patient with a tumour (indicated by the red square in **(d)** using the three-channel external receiver array. The signal contributions from the centre, left, and right coil are indicated in **(a–c)**, respectively, and averaged using WSVD in **(d)**. The spectrum from the tumour **(f)** shows much higher signal of PME as compared to the spectrum obtained contralateral in the tongue **(e)**. Dotted lines in **(a–c)**, show outline of face and tongue as in **(d)**. **(g)** shows a clinical 3-T image and **(h)** is the resection specimen (post-surgery) with the tumour boundary highlighted.

All three elements of the external array contributed to the signal for the ^31^P MRSI of the patient, albeit one element showed overall lower signal intensity (Figures 4a–c). Using WSVD to combine the signals, ^31^P MRSI was successfully obtained and processed, showing spectra distributed all over the tongue (Figure 4d).

When taking a closer look at the spectrum from the tumour area, one can see a substantially elevated signal of PME when compared to a spectrum from a contralateral (healthy) region of the tongue (Figures 4e,f). For reference, a clinical 3T MRI image is shown in Figure 4g, and the excised tongue post-surgery is shown in Figure 4h with the tumour boundary demarcated.

#### Comparison to Internal Loop

When comparing the maximum SNR of the external array with the loop coil on the same subject, about two to three-fold reduced SNR is observed in the array. However, note the substantially increased SNR towards one side of the tongue, close to the jaw muscle. When comparing the SNR of the second subject to the first subject both obtained with the external array, in the second subject, about two-fold more SNR is observed. Overall, the signal levels over the tongue are substantially more uniform with the external array when compared to the loop coil. The array acquires signal from the entire mouth cavity as opposed to the internal coil, which is sensitive only to a portion of the tongue.

### ^31^P Transmit Performance

A flip-angle series using the ^31^P body coil as transmitter and the insert ^31^P coil as receiver indicated a maximum signal when the nominal flip angle was 100° (Figure 2, left) at a TR of 30 s. While shielding between the Tx birdcage and the ^31^P tongue coils might be expected due to the presence of the ^1^H dipoles, this is not seen in practise. The dipoles resonate far off the ^31^P frequency and there is sufficient distance between these two coils (18).

### ^1^H TxRx Dipole Array

The presence of the ^31^P insert coil did not alter the ^1^H B${}_{1}^{+}$ field of the dipole array significantly (i.e., differences of <10%) in a phantom as shown by the comparison between the B${}_{1}^{+}$ maps with or without the presence of the ^31^P insert coil (Figure 2, right).

**Figure 5:**
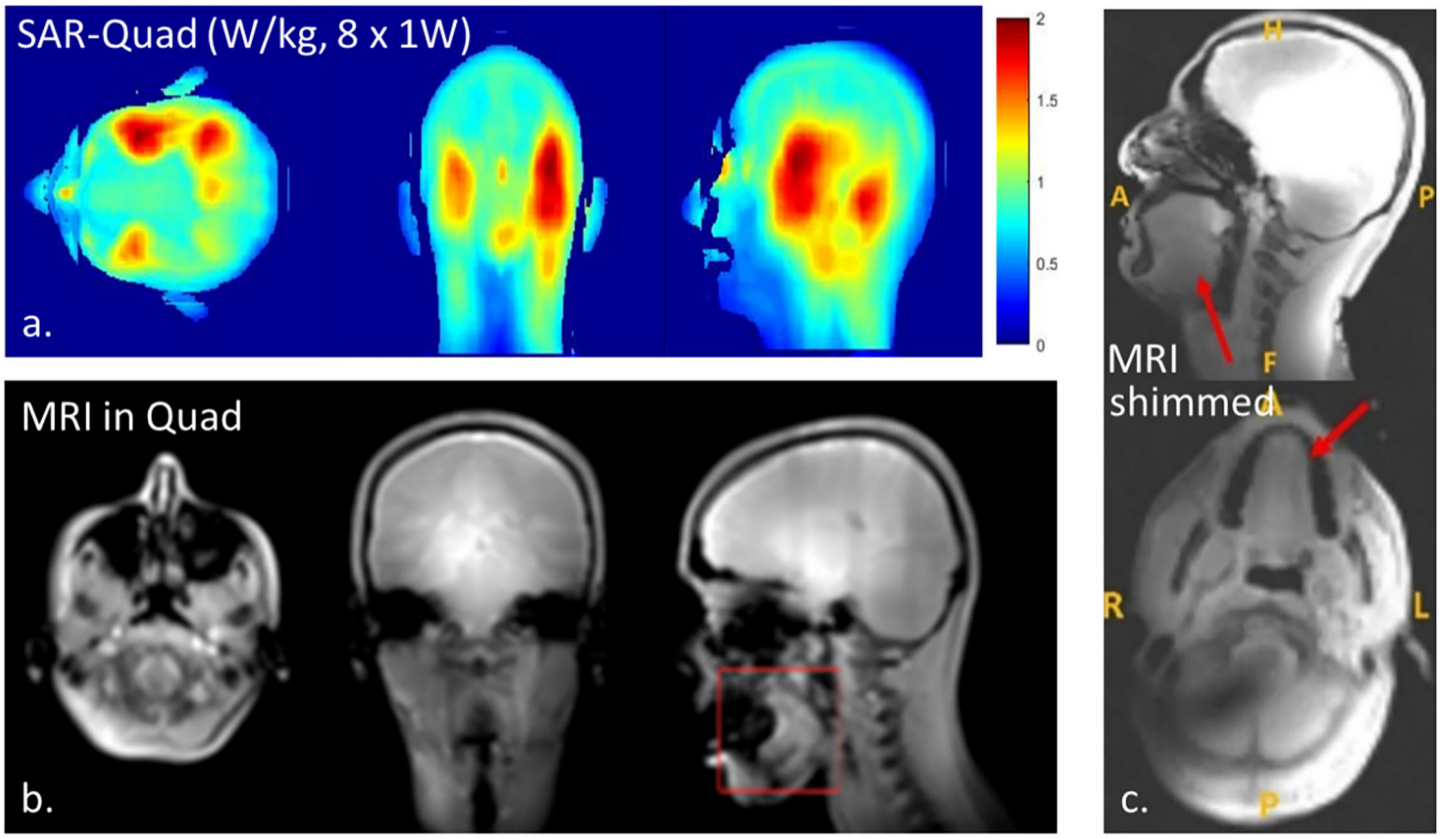
Results with the ^1^H dipole array indicating RF power deposition when driven in quadrature **(a)**, anatomical MRI when driven in quadrature **(b)**, and anatomical MRI when using constructive B${}_{1}^{+}$ interference (RF shimming, **c**). Note that RF shimming in the tongue will result in B${}_{1}^{+}$ shading in certain areas of the brain **(c)**.

RF simulations of the eight-channel dipole array resulted in a peak SAR of 1.94 W/kg and a global SAR of 0.6 W/kg when driven in quadrature at 1 W delivered power for each channel (Figure 5a). When driven at 1 W with an arbitrary phase between the elements, the worst-case local SAR (23) could increase to a maximum of 7.3 W/kg. For all subjects, we have used RF shimming by means of constructive B${}_{1}^{+}$ interference in the tongue. To ensure that we remain within the 10 W/kg peak local SAR in the head (28), we set the maximum allowable power per channel to 1.37 W (1 W × 10 W/kg /7.3 W/kg.

Figure 5 demonstrates anatomical MR images obtained from one of the volunteers using the dipole array driven in quadrature (Figure 5b) and with RF shimming on the tongue (Figure 5c). The anatomical MR images were sufficient for region-of-interest B_0_ shimming. Determining the optimal phase combination for constructive B_1_ shimming took <10 min and was successful in all subjects. While up to third-order B_0_ shimming is available, the B_0_ field uniformity in the tongue could not be better than 40 Hz, expressed as FWHM (full-width at the half of the maximum of the peak) of the offset frequencies of water. Since shimming varies per subject, positioning, tissue coupling, etc. shim values accordingly differ, yet since the shim procedure is automated, we have not noted the actual phase settings.

## Discussion

By combining proton antennas with a traditional full-body birdcage for ^31^P transmit and a dedicated receive array, we present, what is to our knowledge, the first RF coil setup at 7 T for ^31^P MR spectroscopy in the buccal region and tongue. First, we confirmed negligible coupling between the proton array and the phosphorus receive coils to demonstrate safety. The coupling between the two transmit coils has been previously noted to have been insignificant (18). In our study, we have used the SAR setting that allows arbitrary phase setting between channels (which is three-fold more conservative than for quadrature drive).

Next, we have compared the ^31^P performance of a comfortable external receiver array to a close-fitting uncomfortable insert coil. Finally, we have detected a first indication of altered cell proliferation by means of elevated PME levels in the tumour vs. the contralateral healthy area of the tongue in a patient.

Dedicated receiver arrays for MRI of tongue cancer have been presented for proton imaging. Voskuilen et al. (29) could indeed demonstrate a factor of two gain in sensitivity at 3 T in the tongue when comparing their 12-channel array positioned close to the mouth to a commercially built head–neck coil. In addition, inserted ^1^H coils have been proposed at 4 T to perform MRI while the coil is inside the mouth, again showing excellent sensitivity (10). However, they also acknowledged the sub-optimal alignment of the insert coil with respect to the main magnetic field. In our *in vivo* bench test observations that are independent of field orientation, we observed about 6–7 dB loss in B_1_ for each element of the array when compared to a closely positioned insert coil. With optimal signal combination and assuming negligible coupling between elements, based on these bench-top measurements, the sensitivity of the external array is about 80% of the sensitivity of the internal coil, i.e., $\frac{10^{\frac{7}{20}}+10^{\frac{6}{20}}+10^{\frac{7}{20}}}{\sqrt{3}}$. While the sensitivity comparison between coils is difficult to assess in a practical ^31^P MRSI experiment, it can be recognised that the insert coil could provide more SNR, albeit the external array provides a more uniform signal detection over the entire tongue area. While the tip of the tongue is indeed thin, the start of the tongue is relatively thick (see also anatomic MRI). For the tip of the tongue or when tumours are close to the surface, a thin insert coil should provide better SNR. However, in practise, due to the uncomfortable setup, the success rate of a clinical study may be worse. The receive array does not have preamplifier decoupling implemented. Further gains in performance could be obtained by trimming the cable length to optimise it for preamplifier decoupling.

In our study, we used an embedded bore coil as a ^31^P volume transmitter. Previous studies (18, 27, 30) have shown the benefits of a relatively uniform transmit field for ^31^P excitation. It extends the field of view, simplifies RF power calibration, and facilitates the use of Ernst-angle optimised scans for the highest SNR. Making the best out of the compromised magnetic field uniformity in the mouth at 7 T, we could use a relatively short acquisition window that matches the corresponding relatively short T2^*^ to allow a short TR of 57 ms, thus high SNR per unit of time. Moreover, the short TR facilitates the acquisition of many k-points to spatially encode the signal in 3D while being capable of substantial signal averaging of the centre of k-space to match the optimal Hamming weighted acquisition (31).

The magnetic field uniformity was at best 0.13 ppm (40 Hz/298 MHz). Moreover, swallowing and subtle movements during the 10-min MRSI scan can cause more line broadening of the spectra (32). Owing to the large chemical shift dispersion of ^31^P, even in the presence of motion and non-uniform magnetic fields, resonances of PME, Pi, PDE, PCr, and ATP could be well-resolved, which is a distinct advantage over ^1^H spectroscopy. Further improvements in magnetic field shimming and motion correction strategies could be considered when aiming for resolving phosphoethanolamine and phosphocholine peaks of the PME signal (33). For instance, using local shim coils or advanced shimming strategies may improve magnetic field uniformity (34). Even prospective motion correction may be considered to improve the linewidth of the spectra (35).

Recently, other dedicated ^31^P coil setups have been demonstrated for 7 T for brain imaging (19, 20). These state-of-the-art head coils have indicated more than a factor 3 in SNR performance gain when comparing their array with the typical volume coil. While we optimised our MRI setup to acquire the ^31^P MRSI specifically in the tongue, we did incorporate an eight-channel transmit/receive dipole array for proton MRI. Although not fully exploited in this study, phase and amplitude B${}_{1}^{+}$ shimming could significantly improve MRI quality (36). Consequently, the setup may be used for imaging multiple contrast mechanisms in addition to phospholipids and energy metabolism. Recently, Kappert et al. have shown that diffusion-weighted MRI can be used successfully in the tongue (17). Moreover, Athar et al. (37) confirmed that MRI using contrast enhancements could provide 83% accuracy in determining the tumour thickness when compared to histopathology, advising to use MRI for treatment planning in patients with tongue cancer. Potentially, multiple relevant tumour biomarkers like elevated cell proliferation, altered energy metabolism, disrupted perfusion, and hindered diffusion may be studied in patients with tongue cancer to improve treatment decisions.

Our proposed setup was demonstrated to be suitable for metabolic tongue imaging. The proton multi-transceiver system allows for B_1_ shimming to provide proton MRI and B_0_ shimming, the latter being crucial considering the large air pockets of the sinus cavities. However, even though one subject had solid gold fillings, no adverse B_0_ homogeneity artefacts were detected. Similarly, relatively uniform ^31^P excitation could be obtained without the hindrance of the coil since the setup was hidden behind the bore liner of the MRI. Since the ^31^P receive array could be positioned like a face mask, comfortable 3D MRSI could be obtained from the tongue. While the 3D CSI scan itself takes only 10 min, with additional time due to tailor the preparation scans, the scan session, including the proton scans, adds up to a total scan duration similar to clinical MRI scan sessions.

Spatial resolution of the 31P MRSI scan is still compromised to 1.5 cm for the 10-min scan. While higher resolutions can be obtained, it will come at the expense of longer scan times or less SNR. However, it should be noted that despite lower spatial resolution, the effect size in metabolite level can be substantial, so even with partial volume effects, alterations may still indicate tumour extent.

## Conclusion

Here, we have demonstrated a novel coil setup and scan protocols for phosphorus spectroscopy as a means for detecting altered cell proliferation and energy metabolism in tongue tumours. To begin with, we compared the performance of the patient-friendly external three-channel receive array to the ideal case of an intra-oral loop and noticed 80% sensitivity performance of the external array with respect to the inner coil. As an improvement over commercially available head coils, we have used a surface loop array that is inherently decoupled from the proton coil and provides full coverage of the tongue. This setup builds the first steps towards aiding surgery treatment decisions using patient spectroscopy data, even in traditionally hard-to-image anatomic regions such as the mouth.

## Data Availability Statement

The raw data supporting the conclusions of this article will be made available by the authors, without undue reservation.

## Ethics Statement

The studies involving human participants were reviewed and approved by Medisch Ethische Toetsingscommissie (METC) Utrecht; UMC Utrecht. The patients/participants provided their written informed consent to participate in this study.

## Author Contributions

RF: hardware, safety tests, scans for data acquisition, written drafts, and figures. KN: scans for data acquisition, sequence modification, writing, and figures. KK: patient scans and writing. TV: patient scans, sequence modification, and writing. WK: sequence modification, writing, and figures. AR: writing. DK: hardware, writing, and figures. All authors contributed to the article and approved the submitted version.

## Funding

This study was funded by European Union (FET-NICI 801075 and ITN-InspireMed).

## Conflict of Interest

RF was employed by company Ceresensa Inc. The remaining authors declare that the research was conducted in the absence of any commercial or financial relationships that could be construed as a potential conflict of interest.

## Publisher's Note

All claims expressed in this article are solely those of the authors and do not necessarily represent those of their affiliated organizations, or those of the publisher, the editors and the reviewers. Any product that may be evaluated in this article, or claim that may be made by its manufacturer, is not guaranteed or endorsed by the publisher.
